# An Extraordinary Colocalization of Alopecia Areata and Vitiligo

**DOI:** 10.4103/0974-7753.77522

**Published:** 2010

**Authors:** Yuval Ramot, Elena Thomaidou, Alexander Mali, Abraham Zlotogorski

**Affiliations:** Department of Dermatology, Hadassah-Hebrew University Medical Center, Jerusalem, Israel; 1 Trinity College, University of Dublin, Dublin, Ireland; 2 Department of Pathology, Hadassah-Hebrew University Medical Center, Jerusalem, Israel

**Keywords:** Alopecia areata, autoimmunity, vitiligo

## Abstract

Although the association of alopecia areata (AA) and vitiligo occurring in the same patient has been frequently reported in the literature, the colocalization of AA and vitiligo is very rare. We report for the first time an adult patient with anatomic concurrence of AA and vitiligo on the scalp. Even though both AA and vitiligo are thought to have the same underlying pathophysiologic mechanisms, the striking rarity of their colocalization challenges this postulated common pathogenesis, and raises the question if autoimmunity is responsible for only a fraction of AA or vitiligo.

## INTRODUCTION

Alopecia areata (AA) and vitiligo are common dermatological conditions reported to be frequently associated with other autoimmune conditions, particularly thyroiditis, pernicious anemia, and myasthenia gravis, and with each other.[1,2] Nevertheless, although colocalization of these two conditions has been reported already more than 100 years ago,[3] it has been described only three more times since then, and only in children and adolescents,[4–6] making this entity a striking rarity in general and particularly in adults. We present a case of a 60-year-old woman with colocalization of AA and vitiligo, and discuss the possible implications of the infrequency of this entity on AA pathogenesis.

## CASE REPORT

**Figure 1:**
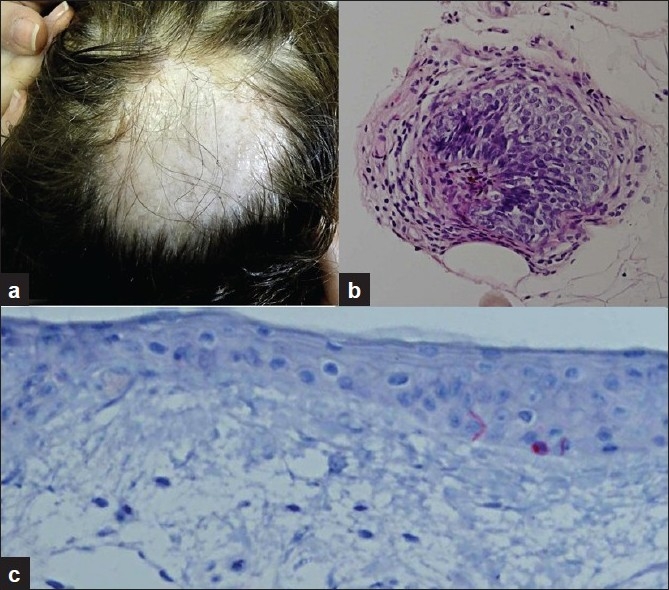
(a) Coexistence of loss of hair and loss of pigment in the patient’s scalp; (b) Histopathology of the scalp lesion demonstrating the presence of an anagen hair follicle with a peribulbar lymphocytic infiltrate, corresponding to the diagnosis of AA; (c) MART-1 immunostaining of the same biopsy, demonstrating almost total loss of melanocytes in the basal layer, with only one or two melanocytes identified in the epidermis, verifying the coexistence of vitiligo in the same region

A 60-year-old woman presented to our clinic with a two-week history of a focal patch of alopecia on her scalp. She had a history of hypothyroidism and hypercholesterolemia, treated with eltroxin and simvastatin, correspondingly. The patient’s daughter was reported to suffer from vitiligo. On physical examination, she had a regularly shaped, well-defined alopecic patch involving the vertex of her scalp. In addition, a depigmented patch was localized almost exactly to the same area as the alopecia [Figure 1a]. She had no other areas of alopecia or depigmentation. A skin biopsy specimen revealed the presence of an anagen hair follicle with a peribulbar lymphocytic infiltrate, confirming the diagnosis of AA [Figure 1b]. In addition, using MART-1 immunostaining, almost total loss of melanocytes was demonstrated in the basal layer, with only one or two melanocytes identified in the epidermis, confirming the coexistence of vitiligo in the same region [Figure 1c].

## DISCUSSION

The pathogenic basis for AA has been for long an enigma, and many theories regarding its elusive etiology have been introduced over time in the medical literature. These include, for example, the genetic, infectious, trophoneurotic, toxic, and endocrinologic hypotheses. However in recent years, the theory that autoimmunity stands at the basis of this disease has gained much support, and is currently considered the prevailing theory, on the basis of both direct and indirect evidence.[7–10] Further support to this theory comes from a recent report based on a large genome-wide association study, which provided evidence that the immune system is involved in this disease, and placed it in the context of other autoimmune diseases.[11] AA has also been reported to be associated with other autoimmune diseases, and particularly with vitiligo, with a frequency of up to 12.5% of AA in vitiligo patients and up to 8% of vitiligo in AA patients.[12,13] Interestingly, the disease initially tends to spare white hairs, and the early hairs to regrow are frequently nonpigmented.[14]

Considering the fact that it was suggested that AA and vitiligo share the same pathogenic pathway, it is surprising that colocalization of these two diseases is not observed more often. One explanation to this apparent infrequency might stem from the fact that antigenically different populations of melanocytes were reported to be present in the epidermis and hair follicle.[15] In addition, it is obvious that the development of an autoimmune disease relies on different combinations of many factors, which include genetic, immune defects, hormonal and environmental factors, according to the ‘mosaic of autoimmunity’ theory.[16] In addition, there is a big inconsistency regarding the prevalence of autoimmune diseases with AA, especially of vitiligo; although some studies show a very high association rate,[13,17] others show association rate as low as 0.3%.[18,19] This apparent high inconsistency in the association of AA with other autoimmune diseases adds to the extremely variable clinical presentations of AA.

The previous few reports on colocalization of AA and vitiligo have highlighted in their discussion the common immunopathologic mechanisms of the two conditions.[4–6] In this study, we claim that the fact that this entity is extremely rare should actually suggest different pathophysiologic mechanisms, and the few reports describing this colocalization can be easily attributed to coincidence alone, or alternatively, that autoimmunity is responsible for only a fraction of AA or vitiligo.

## References

[1] Rezaei N, Gavalas NG, Weetman AP, Kemp EH (2007). Autoimmunity as an aetiological factor in vitiligo. J Eur Acad Dermatol Venereol.

[2] Alexis AF, Dudda-Subramanya R, Sinha AA (2004). Alopecia areata: Autoimmune basis of hair loss. Eur J Dermatol.

[3] Eddowes (1898). Society inteligence. Br J Dermatol.

[4] Adams BB, Lucky AW (1999). Colocalization of alopecia areata and vitiligo. Pediatr Dermatol.

[5] Dhar S, Kanwar AJ (1994). Colocalization of vitiligo and alopecia areata. Pediatr Dermatol.

[6] Yadav S, Dogra S, Kaur I (2009). An unusual anatomical colocalization of alopecia areata and vitiligo in a child, and improvement during treatment with topical prostaglandin E2. Clin Exp Dermatol.

[7] Gilhar A, Paus R, Kalish RS (2007). Lymphocytes, neuropeptides, and genes involved in alopecia areata. J Clin Invest.

[8] Madani S, Shapiro J (2000). Alopecia areata update. J Am Acad Dermatol.

[9] Shapiro J, Dunitz M (2002). Alopecia areata: Pathogenesis, clinical features, diagnosis and practical management. Hair Loss, Principles of Diagnosis and Management of Alopecia.

[10] McElwee KJ, Tobin DJ, Bystryn JC, King LE, Sundberg JP (1999). Alopecia areata: An autoimmune disease?. Exp Dermatol.

[11] Petukhova L, Duvic M, Hordinsky M, Norris D, Price V, Shimomura Y (2010). Genome-wide association study in alopecia areata implicates both innate and adaptive immunity. Nature.

[12] Muller SA, Winkelmann RK (1963). Alopecia areata. An evaluation of 736 patients. Arch Dermatol.

[13] Akay BN, Bozkir M, Anadolu Y, Gullu S (2010). Epidemiology of vitiligo, associated autoimmune diseases and audiological abnormalities: Ankara study of 80 patients in Turkey. J Eur Acad Dermatol Venereol.

[14] Alkhalifah A, Alsantali A, Wang E, McElwee KJ, Shapiro J (2010). Alopecia areata update: Part I. Clinical picture, histopathology, and pathogenesis. J Am Acad Dermatol.

[15] Tobin DJ, Bystryn JC (1996). Different populations of melanocytes are present in hair follicles and epidermis. Pigment Cell Res.

[16] Shoenfeld Y, Isenberg DA (1989). The mosaic of autoimmunity. Immunol Today.

[17] Gopal KV, Rama Rao GR, Kumar YH, Appa Rao MV, Vasudev P (2007). Vitiligo: A part of a systemic autoimmune process. Indian J Dermatol Venereol Leprol.

[18] Liu JB, Li M, Yang S, Gui JP, Wang HY, Du WH (2005). Clinical profiles of vitiligo in China: An analysis of 3742 patients. Clin Exp Dermatol.

[19] Schallreuter KU, Lemke R, Brandt O, Schwartz R, Westhofen M, Montz R (1994). Vitiligo and other diseases: Coexistence or true association. Hamburg study on 321 pateints?. Dermatology.

